# 

**Published:** 1879-04

**Authors:** 


*CONTENTS.*

| | Page |
|---|---|
| The Idea of Wealth . . . | 777 |
| Manners . . . . . . . . . | 779 |
| Without Medicine . . . . | 780 |
| Debtor and Creditor . . . | 789 |
| Restless Nights . . . . . . | 801 |
| Leaving Home . . . . . | 802 |
| Book Notices . . . . . . . . | 803 |

